# Genome Sequence of Brevibacillus brevis HK544, an Antimicrobial Bacterium Isolated from Soil in Daejeon, South Korea

**DOI:** 10.1128/MRA.00417-21

**Published:** 2021-08-05

**Authors:** Bomin Kim, Yeong Seok Kim, Jae Woo Han, Gyung Ja Choi, Hun Kim

**Affiliations:** a Center for Eco-friendly New Materials, Korea Research Institute of Chemical Technology, Daejeon, South Korea; b Department of Medicinal Chemistry and Pharmacology, University of Science and Technology, Daejeon, South Korea; University of Arizona

## Abstract

The Brevibacillus brevis HK544 strain, which was isolated from soil, exhibited antimicrobial activity against plant pathogens such as Botrytis cinerea, Phytophthora infestans, and Erwinia amylovora. Here, we report the draft genome sequence of the B. brevis HK544 strain, which consists of one circular chromosome of 6,486,246 bp with a GC content of 47.3%.

## ANNOUNCEMENT

The genus *Brevibacillus* phylogenetically belongs to the family *Paenibacillaceae*, which normally is found in a variety of environments such as the intestinal tracts of animals, seawater, and soil (1). To date, more than 22 species have been assigned taxonomically to the genus *Brevibacillus* (2). Considering that various *Brevibacillus* species have been recognized as rich sources of antimicrobial peptides, several strains of *Brevibacillus* species have been studied for use as biocontrol agents against plant pathogens (3–5). In the current study, the Brevibacillus brevis HK544 strain, which was originally isolated from soil, exhibited antifungal and antibacterial activities against plant pathogens such as Botrytis cinerea, Phytophthora infestans, and Erwinia amylovora (6).

To isolate the HK544 strain, soil samples were collected at a 10-cm depth from forests at the Korea Research Institute of Chemical Technology (Daejeon, South Korea). One gram of each soil sample was suspended in 9 ml of distilled water, and these suspensions were serially diluted and streaked on a nutrient agar plate. Based on the agar disk diffusion method (7), isolates showing antifungal activity were selected as candidates. In addition to antifungal activity, the culture filtrate of the selected HK544 strain was investigated for antibacterial activity by broth microdilution assays using the 2-fold serial dilution method described for the modified CLSI M38-A method (8). The HK544 strain was grown in tryptic soy broth (TSB) (BD, Sparks, MD, USA) at 30°C for 48 h, and the HK544 cells were suspended in 20% glycerol solution and kept at −80°C. A single colony from tryptic soy agar (TSA) was grown in TSB (BD) at 30°C for 48 h, and the genomic DNA of the HK544 strain was extracted using QIAamp genomic DNA kits (Qiagen, Hilden, Germany), following the manufacturer’s instructions. The purity and concentration of the genomic DNA were determined with a 2100 Bioanalyzer system (Agilent Technologies, Palo Alto, CA, USA).

The whole genome of the HK544 strain was sequenced with a 20-kb SMRTbell library (Pacific Biosciences [PacBio] DNA/polymerase binding kit P6) on the RS II sequencing platform (PacBio) using C4 chemistry with eight single-molecule real-time (SMRT) cells at Macrogen (Seoul, South Korea) (9). A total of 122,001 PacBio subreads (*N*_50_, 13,458 bp; mean subread length, 8,688 bp) were generated, with 163-fold coverage of the genome. The cleaned reads were assembled *de novo* using Canu v1.7 (10), and the result was polished with error correction by Pilon v1.23 (11). All tools were run with default parameters unless otherwise specified. The whole genome of the HK544 strain features a single circular chromosome that is 6,486,246 bp in length, with a GC content of 47.3%, and no plasmids were detected.

Genome annotation was performed with the NCBI Prokaryotic Genome Annotation Pipeline (PGAP) v5.1 (12). In total, 5,766 coding DNA regions were identified, with 127 tRNAs, 41 rRNAs, 5 noncoding RNAs, and 194 pseudogenes. The 16S rRNA gene sequence alignment on the EzTaxon server (http://www.ezbiocloud.net) revealed that the HK544 strain belongs to the genus *Brevibacillus* and is most closely related to B. brevis NBRC 15304^T^ (GenBank accession number AB271756) and Brevibacillus porteri NRRL B-41110^T^ (GenBank accession number PXZO01000094), with similarities of 99.86 and 99.73%, respectively. Additionally, when the average nucleotide identity (ANI) with the currently available genome sequences of *Brevibacillus* spp. was calculated using OAT v0.93 (13), the HK544 strain showed very high (>93.8%) ANI values with respect to B. brevis DZQ7 (GenBank accession number CP030117.1) and Brevibacillus formosus NF2 (GenBank accession number CP018145.1) at the genome level.

Considering that *Brevibacillus* spp. produce various bioactive peptides, the B. brevis HK544 genome also contains several genes involved in antimicrobial activity. Analysis with antiSMASH v4.2 revealed that the HK544 genome contains 14 gene clusters for nonribosomal peptide synthetases and polyketide synthases and for bacteriocins, terpenes, siderophores, and lanthipeptides (14). The genome sequence presented in this work enables us to gather more insights into the genetic foundation for the biocontrol mechanisms against plant pathogens and might be a valuable resource for the development of biopesticides.

### Data availability.

The genome sequence of the B. brevis HK544 strain has been deposited in the NCBI GenBank database under accession number CP042161. The BioProject, BioSample, and SRA accession numbers are PRJNA556321, SAMN12347649, and SRR14827090, respectively.

## References

[1] Shida O, Takagi H, Kadowaki K, Komagata K. 1996. Proposal for two new genera, *Brevibacillus* gen. nov. and *Aneurinibacillus* gen. nov. Int J Syst Bacteriol 46:939–946. doi:10.1099/00207713-46-4-939. 8863420

[2] Sanket R, Nafisa P, Dhruti A. 2020. *Brevibacillus*, p 149–167. *In* Amaresan N, Kumar MS, Annapurna K, Kumar K, Narayanan S (ed), Beneficial microbes in agro-ecology, 1st ed. Academic Press, Cambridge, MA.

[3] Jianmei C, Bo L, Zheng C, Huai S, Guohong L, Cibin G. 2015. Identification of ethylparaben as the antimicrobial substance produced by *Brevibacillus brevis* FJAT-0809-GLX. Microbiol Res 172:48–56. doi:10.1016/j.micres.2014.11.007. 25542595

[4] Haggag WM. 2008. Isolation of bioactive antibiotic peptides from *Bacillus brevis* and *Bacillus polymyxa* against *Botrytis* grey mould in strawberry. Biocontrol Sci Technol 41:477–491. doi:10.1080/03235400600833704.

[5] Hou Q, Wang C, Hou X, Xia Z, Ye J, Liu K, Liu H, Wang J, Guo H, Yu X, Yang Y, Du B, Ding Y. 2015. Draft genome sequence of *Brevibacillus brevis* DZQ7, a plant growth-promoting rhizobacterium with broad-spectrum antimicrobial activity. Genome Announc 3:e00831-15. doi:10.1128/genomeA.00831-15. 26294619 PMC4543497

[6] Kim H, Choi GJ, Han JW, Choi YH. 2020. Composition for controlling plant diseases using *Brevibacillus brevis* HK544. South Korean patent 10–2020-0077212.

[7] Arikan S, Paetznick V, Rex JH. 2002. Comparative evaluation of disk diffusion with microdilution assay in susceptibility testing of caspofungin against *Aspergillus* and *Fusarium* isolates. Antimicrob Agents Chemother 46:3084–3087. doi:10.1128/AAC.46.9.3084-3087.2002. 12183278 PMC127447

[8] Espinel-Ingroff A, Fothergill A, Ghannoum M, Manavathu E, Ostrosky-Zeichner L, Pfaller M, Rinaldi M, Schell W, Walsh T. 2005. Quality control and reference guidelines for CLSI broth microdilution susceptibility method (M 38-A document) for amphotericin B, itraconazole, posaconazole, and voriconazole. J Clin Microbiol 43:5243–5246. doi:10.1128/JCM.43.10.5243-5246.2005. 16207990 PMC1248457

[9] Tombácz D, Csabai Z, Oláh P, Balázs Z, Likó I, Zsigmond L, Sharon D, Snyder M, Boldogkői Z. 2016. Full-length isoform sequencing reveals novel transcripts and substantial transcriptional overlaps in a herpesvirus. PLoS One 11:e0162868. doi:10.1371/journal.pone.0162868. 27685795 PMC5042381

[10] Chin CS, Alexander DH, Marks P, Klammer AA, Drake J, Heiner C, Clum A, Copeland A, Huddleston J, Eichler EE, Turner SW, Korlach J. 2013. Nonhybrid, finished microbial genome assemblies from long-read SMRT sequencing data. Nat Methods 10:563–569. doi:10.1038/nmeth.2474. 23644548

[11] Walker BJ, Abeel T, Shea T, Priest M, Abouelliel A, Sakthikumar S, Cuomo CA, Zeng Q, Wortman J, Young SK, Earl AM. 2014. Pilon: an integrated tool for comprehensive microbial variant detection and genome assembly improvement. PLoS One 9:e112963. doi:10.1371/journal.pone.0112963. 25409509 PMC4237348

[12] Tatusova T, DiCuccio M, Badretdin A, Chetvernin V, Nawrocki EP, Zaslavsky L, Lomsadze A, Pruitt KD, Borodovsky M, Ostell J. 2016. NCBI Prokaryotic Genome Annotation Pipeline. Nucleic Acids Res 44:6614–6624. doi:10.1093/nar/gkw569. 27342282 PMC5001611

[13] Lee I, Kim YO, Park SC, Chun J. 2016. OrthoANI: an improved algorithm and software for calculating average nucleotide identity. Int J Syst Evol Microbiol 66:1100–1103. doi:10.1099/ijsem.0.000760. 26585518

[14] Weber T, Blin K, Duddela S, Krug D, Kim HU, Bruccoleri R, Lee SY, Fischbach BA, Müller R, Wohlleben W, Breitling R, Takano E, Medema MH. 2015. antiSMASH 3.0: a comprehensive resource for the genome mining of biosynthetic gene clusters. Nucleic Acids Res 43:W237–W243. doi:10.1093/nar/gkv437. 25948579 PMC4489286

